# NTRK1-fusion as an acquired resistance mechanism in EGFRex19 mutated NSCLC: a case report

**DOI:** 10.3389/fphar.2025.1571777

**Published:** 2025-08-06

**Authors:** Jielin Li, Yunyun Shi, Mengge Zheng, Chenkang Yang, Hong Gao, Xiaoling Li

**Affiliations:** ^1^ Department of Thoracic Internal Medicine, Cancer Hospital of China Medical University, Liaoning Cancer Hospital and Institute, Shenyang, Liaoning, China; ^2^ Genetron Health (Beijing) Co. Ltd., Beijing, China

**Keywords:** EGFR-mutated NSCLC, NTRK-fusion, larotrectinib, resistance, case report

## Abstract

**Introduction:**

A 73-year-old Chinese woman with no smoking history was diagnosed with stage IV adenocarcinoma of the lung in August 2020, presenting with left chest pain and multiple lung lesions.

**Patient concerns:**

The patient experienced chest pain for 2 months before diagnosis. Initial CT scans revealed multiple lung nodules, enlarged lymph nodes, and pleural effusion.

**Diagnosis:**

The diagnosis was confirmed as advanced NSCLC the left upper lobe of the lung with specific genetic alterations, including EGFR 19del, EGFR amplification, and TPR-NTRK1 fusion, through molecular testing.

**Interventions:**

Prior treatments included the first-line therapy gefitinib (250 mg/day) administered from September 2020 to June 2021, targeting the EGFR 19del mutation, achieving a partial response (PR). The second-line therapy osimertinib (80 mg/day) was administered from July 2021 to January 2022, targeting EGFR 19del and T790M mutations, with a progression-free survival (PFS) of approximately 7 months. The third-line therapy almonertinib, another third-generation EGFR-TKI, was administered from January 2022 to March 2022, but the response was poor, leading to further progression. After identifying NTRK fusion and EGFR amplification, the patient was administered larotrectinib as third-line treatment. Prior treatments included targeted therapies and chemotherapy.

**Outcomes:**

Despite multiple lines of targeted therapy, the patient experienced rapid disease progression at several points, highlighting the challenges in managing NSCLC with complex genetic alterations.

**Conclusion:**

This case underscores the importance of ongoing molecular testing and the potential need for combination therapies in managing advanced NSCLC with resistance to multiple targeted treatments. The current treatment with camrelizumab combined with chemotherapy shows promise, but further monitoring is necessary.

## Introduction

Lung cancer is the most common cause of cancer-related death. Non-small cell lung cancer (NSCLC) accounts for approximately 85% of primary lung tumors with 5-year overall survival (OS) of less than 20% for newly diagnosed patients (Brahmer et al., 2018). The median OS from patients with initial metastatic NSCLC cancer not previously treated with systemic therapy was <1 year (Simeone et al., 2019).

The mutational landscape of NSCLC reveals distinct molecular subtypes, with EGFR mutations being the most prevalent driver alterations, particularly in Asian populations (Kobayashi et al., 2023; Zhou et al., 2021). These mutations, particularly exon 19 deletions and L858R substitutions, constitutively activate the EGFR pathway, making them prime targets for tyrosine kinase inhibitors (TKIs) (Huang et al., 2022). Molecular targeted therapeutics, such as tyrosine kinase inhibitors (TKIs) targeting the EGFR/ALK, have greatly improved the survival of NSCLC patients (Wang et al., 2021). Recent advances in genomic profiling have identified novel targetable alterations beyond EGFR/ALK, including NTRK gene fusions, RET rearrangements, and KRAS G12C mutations. The development of selective inhibitors against these targets (e.g., larotrectinib for NTRK and selpercatinib for RET) has shown unprecedented efficacy in clinical trials, offering new hope for previously untreatable NSCLC subgroups (Cocco et al., 2018).

The NTRK gene family consists of NTRK1, NTRK2, and NTRK3, which encode the TRK family proteins TRKA, TRKB, and TRKC, respectively. Structurally, TRK family proteins consist of an extracellular ligand-binding region, transmembrane region, and intracellular tyrosine kinase region (Huang and Reichardt, 2001). Normally, TRK family proteins are predominantly expressed in neural tissues and play a crucial role in neural cell differentiation and survival, as well as in axon and dendrite formation, embryonic development, and maintenance of nervous system function. After binding to the corresponding ligands, TRK receptor proteins dimerize and activate multiple downstream signaling pathways, including the MAPK, PI3K/AKT, and PLC-γ pathways. These three pathways play important roles in cell function (Amatu et al., 2019).

We present the case of a female patient with advanced NSCLC (stage IV). She received gefitinib (EGFR 19del) and osimertinib/almonertinib (EGFR 19del/T790M) as first- and second-line therapies, respectively. NGS sequencing of the patient’s biopsy tissue after second-line treatment were performed. The rebiopsy showed maintained EGFR 19del and EGFR amplification, but also revealed the acquisition of a TPR-NTRK1 fusion. Larotrectinib is used as a third-line treatment, but its efficacy is poor. In accordance with the CARE reporting checklist, we present the following case.

## Case report

**FIGURE 1:**
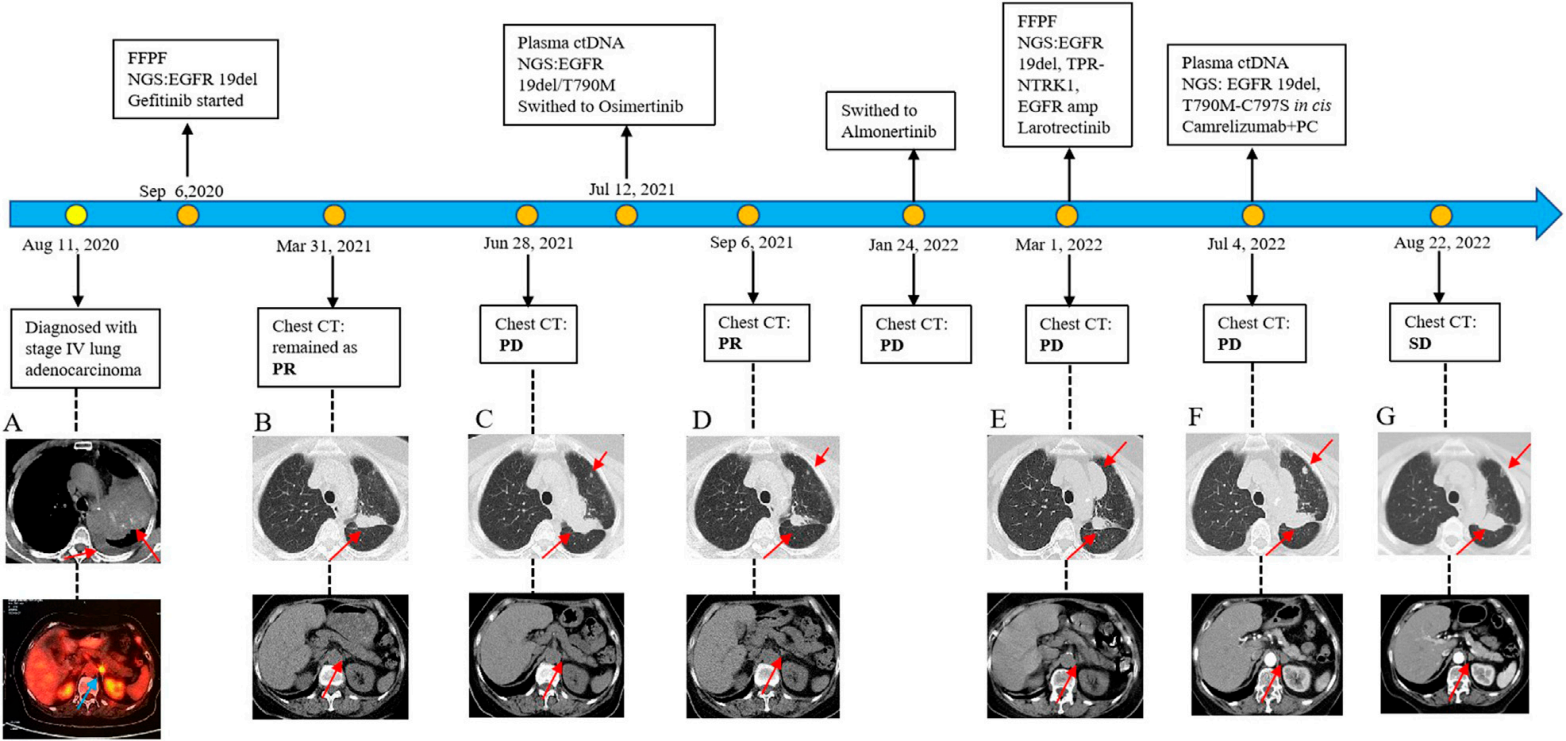
Diagram of the patient’s diagnosis and treatment course. **(A)** Initial CT image at diagnosis showing multiple lung lesions. **(B)** Partial response (PR) to gefitinib. **(C)** Progression of disease (PD) on gefitinib. **(D)** PR to osimertinib. **(E)** PD on almonertinib. **(F)** PD on larotrectinib. **(G)** Stable disease (SD) achieved with camrelizumab combined with pemetrexed and cisplatin (PC). Genomic sequencing revealed an NTRK1 fusion (exons 2–19 of NTRK1) as a key resistance mechanism.

In August 2020, a 73-year-old Chinese woman with no smoking history presented to the hospital with left chest pain that had lasted for 2 months. The computed tomography (CT) scan revealed several lesions in the left upper lobe of the lung, along with additional findings such as multiple nodules, enlarged mediastinal lymph nodes, and pleural effusion (Figure 1A). The patient underwent bronchoscopy, during which a biopsy was obtained from a lesion in the left upper lobe of the lung. Immunohistochemistry and molecular testing were performed on the biopsy. The diagnosis of stage IV adenocarcinoma (T4N3M1c) was established based on CT findings, while the bronchoscopy provided histopathological confirmation. For molecular profiling, next-generation sequencing (NGS) using an 825-gene panel (Genetron Health; Beijing, China) was performed on the patient’s lung biopsy sample and revealed EGFR 19del (p.L747_T751del) with a variant allele frequency (VAF) of 64.7%. NGS also showed microsatellite stability (MSS) and low tumor mutational burden (TMB) level of 0.94 mutations per Megabase (Mb). The other potential cancer-related mutations are listed in Table 1. In addition, immunohistochemistry of programmed cell death ligand-1 (PD-L1) revealed a tumor proportion score (TPS) was 1%–2%. In September 2020, the patient was treated with gefitinib (250 mg/day) as first-line targeted therapy. In March 2021, a review of the patient’s CT scans showed that the disease remained in partial response (PR) (Figure 1B). Minimal side effects, including diarrhea and rash, were observed.

**TABLE 1:** Tissue and ctDNA NGS results.

			Values (Mutation frequency/copy number/status)			
Gene/Biomarker	cHGVS	pHGVS				
			Biopsy tissue	ctDNA,	Biopsy tissue	ctDNA,
			Aug. 2020	Jul. 2021	Mar. 2022	Jul. 2022
AKT2	c.508C>T	p.R170W	ND	ND	ND	0.30%
EGFR	c.2240_2254del	p.L747_T751del	64.70%	8.80%	52.30%	3.40%
EGFR	Amplification	–	2.5	ND	8.5	ND
EGFR	c.2369C>T	p.T790M	ND	2.20%	ND	1.90%
EGFR	c.2389T>A	p.C797S	ND	ND	ND	0.10%
GNAS	c.1738C>T	p.R580W	12.10%	1.40%	ND	ND
GRM3	c.304G>A	p.D102N	ND	ND	ND	0.60%
HNF4A	c.484G>A	p.V162I	ND	1.00%	ND	0.40%
PAK7	c.1301C>T	p.A434V	ND	ND	ND	0.30%
RBM10	c.2354_2357del	p.D785Afs*81	7.20%	1.70%	ND	0.60%
SMAD4	c.1486C>T	p.R496C	ND	0.10%	ND	ND
SMARCB1	c.641C>T	p.T214M	ND	0.70%	ND	ND
TET2	c.4821del	p.Y1608Ifs*2	ND	ND	ND	0.50%
TET2	c.4886_4889del	p.P1629Hfs*65	ND	ND	ND	0.30%
TP53	c.743G>A	p.R248Q	12.80%	1.10%	73.30%	1.00%
TPR-NTRK1	–	–	ND	ND	5.10%	ND
MSI status	–	–	MSS	ND	MSS	ND
TMB (mutations/Mb)	–	–	0.94	3.23	ND	3.23
PD-L1 (TPS)	–	–	1%–2%	ND	<1%	ND

ctDNA, circulating tumor DNA; NGS, next-generation sequencing; ND, not detected; cHGVS, Coding DNA, reference sequence based on Human Genome Variation Society nomenclature; pHGVS, Protein reference sequence based on Human Genome Variation Society nomenclature; TMB, tumor mutational burden; MSI, microsatellite instability; PD-L1, programmed cell death ligand 1.

**FIGURE 2:**
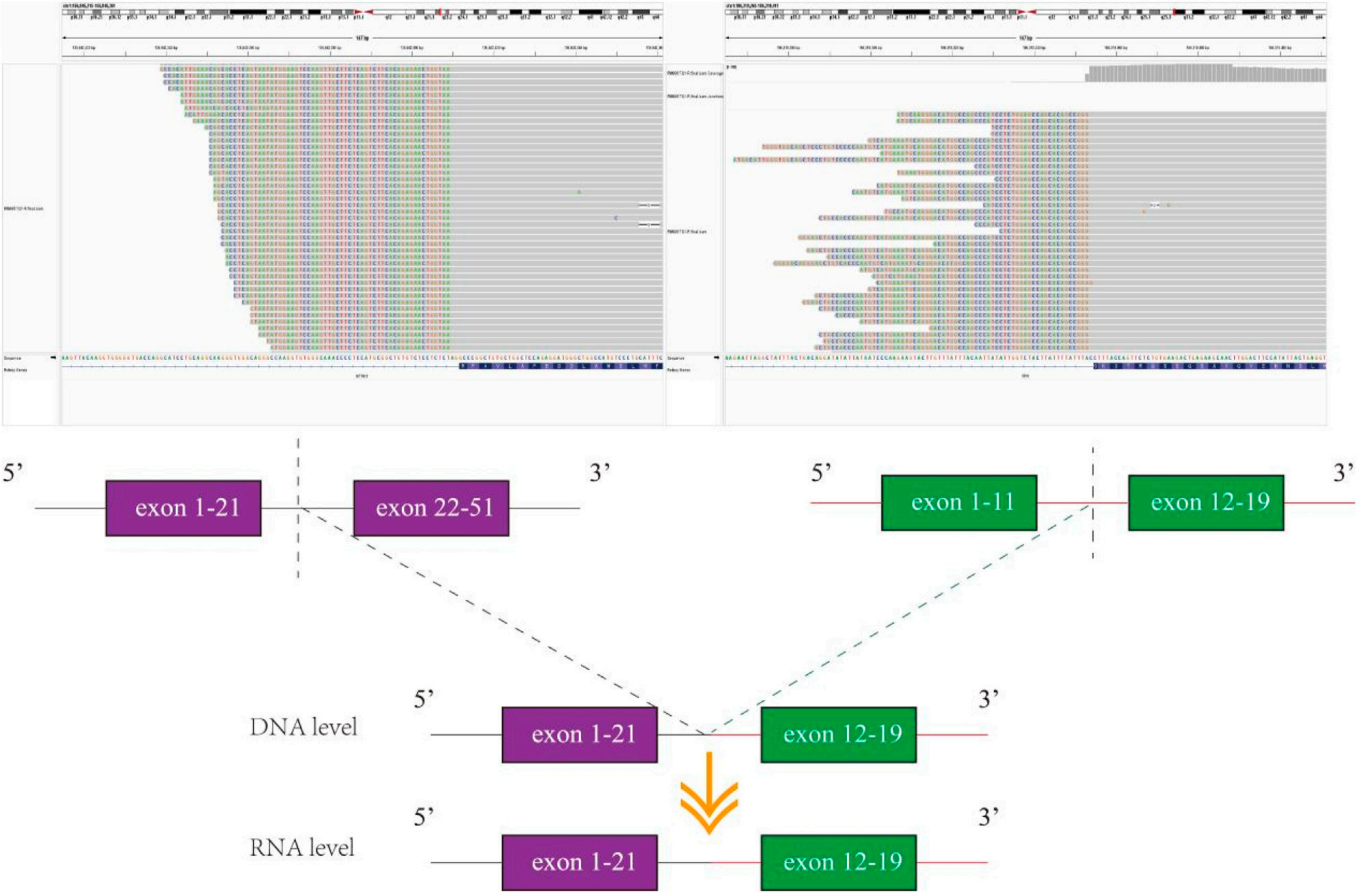
Genomic fusion of exons 1–21 of TPR to exons 12–19 of NTRK1.522656.

Unfortunately, after 10 months of continuous treatment (28 June 2021), Chest CT scans showed an increased pulmonary lesion in the left upper lobe, enlarged nodules in the left pulmonary and left pleural, and decreased effusion in the left pleural, which was evaluated as progressive disease (PD) (Figure 1C). Owing to the possibility of the patient being resistant to gefitinib and reluctance to undergo a biopsy, a plasma sample was obtained for NGS using an 825-gene panel (Genetron Health; Beijing, China). The NGS result revealed the retention of EGFR 19 del (p.L747_T751del) (VAF:8.8%) and the emergence of T790M (VAF:2.2%) (Table 1). Blood-based tumor mutational burden (bTMB) was 3.23 mutations/Mb. Following a positive test result for circulating tumor DNA (ctDNA), the patient’s second-line treatment was switched to osimertinib (80 mg/day) in July 2021. The best objective response (OR) was PR, as determined by CT scan results from September 2021 (Figure 1D). In January 2022, Chest CT scans indicated disease progression as the lesions near the aortic arch and the left pulmonary nodules were observed to have increased in size compared to earlier images, indicating a progression-free survival (PFS) of approximately 7 months were achieved during second-line treatment with osimertinib before disease progression was observed. Considering the excellent performance of the third-generation EGFR-TKI almonertinib in the Chinese population, treatment was switched to almonertinib despite its similar mechanism of action to osimertinib. This decision was based on clinical judgment and the limited availability of alternative targeted therapies at that time. Regrettably, the response remained unsatisfactory, and the tumor continued to progress (Figure 1E) after treatment for over 2 months. In March 2022, an ultrasound-guided lung puncture biopsy was performed in the interventional department, and IHC showed non-small cell carcinoma, consistent with adenocarcinoma, with a few cells weakly expressing neuroendocrine markers. Capture-based targeted sequencing of lung puncture samples after almonertinib treatment was performed using a panel comprising 119 cancer-related genes (Genetron Health; Beijing, China). The results indicated a MSS tumor with EGFR 19del (p.L747_T751del) (VAF: 52.3%), TPR-NTRK1 fusion (VAF: 5.1%), and EGFR amplification (8.5 copies) (Table 1). TPR-NTRK1 gene fusion alterations were verified by RNA sequencing (Figure 2). Therefore, larotrectinib (100 mg bid) was used as third-line treatment for this patient from 19 March 2022. Unfortunately, in July 2022, a chest CT scan revealed that the disease progressed again, with an enlarged space-occupying area of the left upper lobe of the lung (TPR-NTRK1 fusion lesion), increased left pulmonary nodules, a small amount of pleural effusion on the left side, and a thickened left adrenal gland (Figure 1F). Plasma-based NGS using a panel of 825 cancer-related genes (Genetron Health; Beijing, China) indicated the presence of EGFR 19del (p.L747_T751del) (VAF: 3.4%), EGFR T790M-cis-C797S (VAF: 0.1%), and EGFR T790M (VAF: 1.9%) (Table 1). Blood-based TMB was 3.23 mutations/Mb. The C797S mutation has been reported to be responsible for osimertinib resistance (Zeng et al., 2022), and there is a lack of follow-up treatment recommendations. According to previous case reports (Song et al., 2022), the combination of PD-1 monoclonal antibody and platinum-containing dual-drug chemotherapy may be an effective treatment strategy for patients with EGFR T790M-cis-C797. Therefore, since July 2022, the patient has been receiving camrelizumab combined with pemetrexed and cisplatin. As of November 2022, the patient had taken the drugs for two cycles and was in a stable condition (Figure 1G). Follow-up revealed that the patient passed away on 15 June 2024.

## Discussion

The emergence of acquired resistance represents a fundamental challenge in the management of EGFR-mutant non-small cell lung cancer (NSCLC) treated with tyrosine kinase inhibitors (TKIs). The spectrum of resistance mechanisms to EGFR-TKIs encompasses several well-characterized molecular pathways. The most common involves the development of secondary EGFR mutations, particularly T790M following first-generation TKI treatment (Yun et al., 2008) and C797S after third-generation inhibitors (Kagawa et al., 2023). Alternative resistance pathways frequently involve activation of bypass signaling tracks, including MET amplification (Jakobsen et al., 2017), HER2 amplification (Takezawa et al., 2012), and various RAS-RAF pathway alterations. Phenotypic plasticity, particularly epithelial-mesenchymal transition (EMT), constitutes another clinically relevant resistance pattern that promotes tumor cell survival and dissemination (Weng et al., 2019).

Recent advances in comprehensive molecular profiling have identified kinase fusions as an emerging category of resistance mechanisms in EGFR-mutant NSCLC. While RET and BRAF fusions have been documented in TKI-resistant cases (Schrock et al., 2018; Wang et al., 2022), chromosomal rearrangements involving NTRK1 are rare in NSCLC, with a frequency ranging from 0.1% to 3.3% (Liu et al., 2022). The present case provides compelling evidence for this novel resistance paradigm, demonstrating acquisition of a TPR-NTRK1 fusion alongside persistent EGFR 19del and EGFR amplification following progression on sequential EGFR-TKIs. This molecular profile suggests a complex evolutionary pattern under therapeutic pressure, where both EGFR-dependent and NTRK1 fusion-driven clones coexist as parallel resistance mechanisms.

**FIGURE 3:**
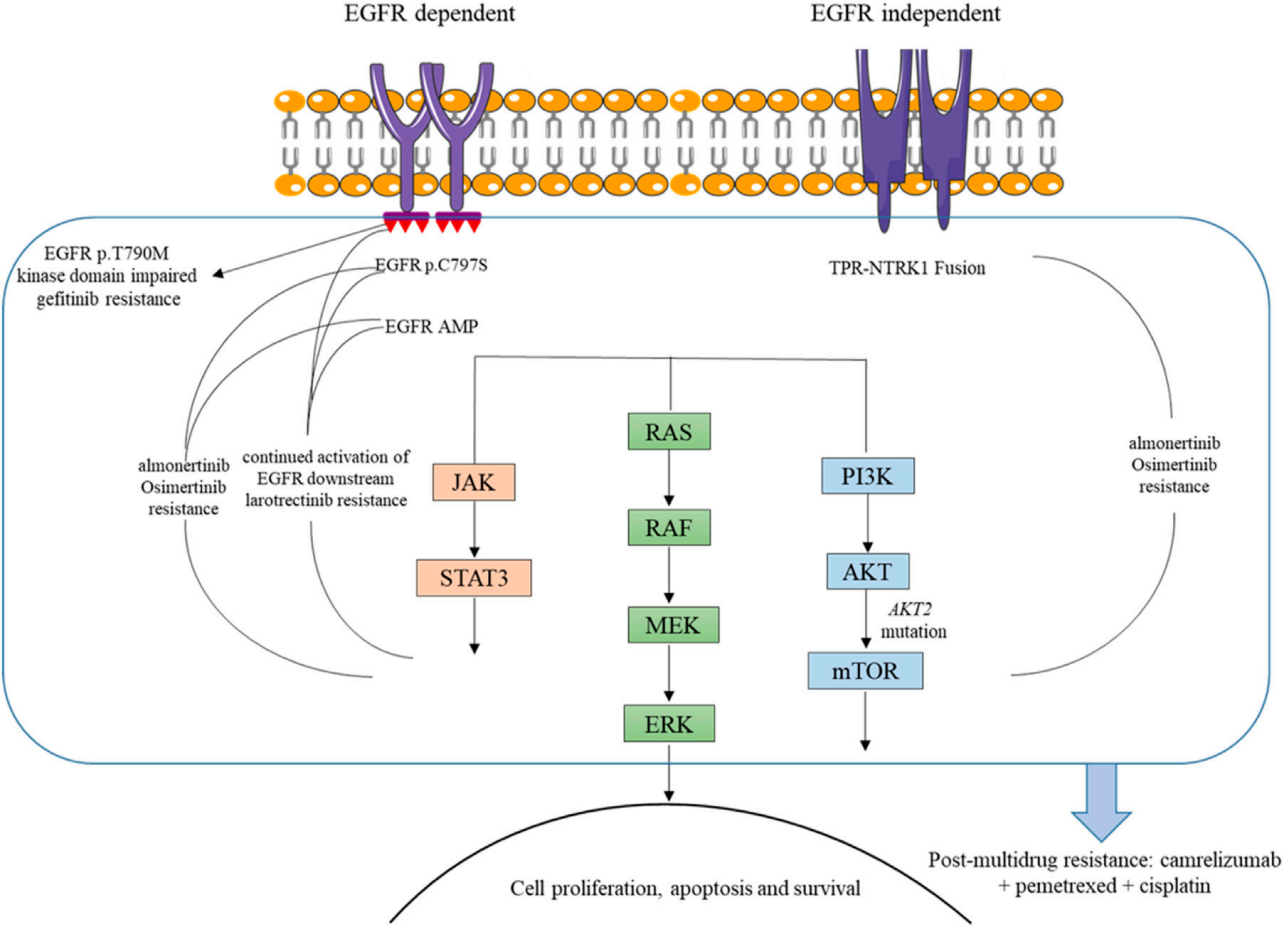
Mechanisms of resistance to TKIs and basis for camrelizumab combination in the presented case.

NTRK gene fusions have been identified as oncogenic drivers for a variety of adult and pediatric tumors, and TRKs are emerging as important targets for cancer therapeutics. While TRK proteins become constitutively activated or overexpressed when NTRK genes fuse with other partner genes, leading to sustained activation of multiple downstream signaling pathways, including RAS/MAPK and PI3K/AKT, thereby facilitating tumor cell proliferation and metastasis (Figure 3) (Liu et al., 2022; Jiang et al., 2021). Entrectinib or larotrectinib has been approved by the FDA for the treatment of advanced or metastatic solid tumors carrying NTRK gene fusions, owing to their ability to inhibit the aberrant MAPK and PI3K/AKT signaling pathways (Haratake and Seto, 2021). While patients with tumors harboring NTRK fusions typically exhibit a sustained response to TRK-targeted therapy, most patients inevitably develop acquired resistance. Previous studies have suggested that reactivation of the RAS/RAF/MEK/ERK signaling pathway may be responsible for resistance to NTRK inhibitors (Vaishnavi et al., 2020).

This patient carried EGFR 19del and EGFR amplification as well as TPR-NTRK1 fusion before third-line treatment. Notably, EGFR 19del and EGFR T790M were detected in NGS plasma samples after larotrectinib treatment. In NSCLC, EGFR mutations lead to excessive activation of downstream pro-survival signaling pathways, thereby promoting tumorigenesis (Hsu et al., 2019). Considering all these, we speculated that the continued activation of EGFR downstream signaling pathways, such as RAS/RAF/MEK/ERK, may be one reason for the unsatisfactory therapeutic effect of larotrectinib in this patient (Figure 3). However, further preclinical studies and larger clinical cohorts are warranted to validate this hypothesis and elucidate the underlying resistance mechanisms.

Furthermore, Cocco et al. reported a patient with entrectinib resistance due to acquired MET amplification, whose disease was controlled by treatment with selitrectinib and the multikinase MET inhibitor crizotinib, and the MET amplification and NTRK fusion previously detected in circulating cell-free DNA (cfDNA) disappeared (Cocco et al., 2019). Furthermore, Lin et al. (2021) reported a case in which a patient had a Notch2-NTRK1 fusion and EGFR L858R detected after progression to osimertinib treatment and was subsequently treated with larotrectinib in combination with osimertinib with significant clinical improvement. Therefore, the absence of concurrent EGFR-TKI therapy during larotrectinib treatment likely contributed to the rapid disease progression observed in this case. This highlights the potential importance of dual inhibition strategies for managing patients with coexisting EGFR mutations and NTRK fusions, as evidenced by prior studies showing clinical benefit from such approaches (Lin et al., 2021). Future studies and clinical trials are needed to explore the efficacy and safety of combined EGFR and TRK inhibitors in overcoming resistance and improving outcomes for this subset of patients.

In summary, this case report demonstrates that larotrectinib has an unsatisfactory therapeutic effect in NSCLC patients with TPR-NTRK1 fusion and EGFR mutation, probably due to the persistent activation of downstream signaling pathways such as RAF/MEK/ERK. From our case and our point of view, the combination of a TRK inhibitor and an EGFR inhibitor may be an effective treatment option for NSCLC patients with TPR-NTRK1 fusion and EGFR mutations. However, more evidence and clinical trials are needed to evaluate the causes of larotrectinib resistance and the efficacy and safety of the combination of TRK inhibitors and EGFR inhibitors in the treatment of NSCLC patients harboring TPR-NTRK1 fusion and EGFR mutations. It is our hope that this case report can enrich the knowledge regarding the treatment and clinical outcomes of patients with this unusual condition and serve as a reference for similar future cases.

## Data Availability

The original contributions presented in the study are included in the article/supplementary material, further inquiries can be directed to the corresponding author.
